# Dose escalation in pentylenetetrazol kindling detects differences in chronic seizure susceptibility

**DOI:** 10.1016/j.eplepsyres.2026.107755

**Published:** 2026-02-13

**Authors:** Mitchell B. Moyer, Jenna Langbein, Orest Tsymbalyuk, Darrian McAfee, Chixiang Chen, Muznabanu Bachani, Volodymyr Gerzanich, J. Marc Simard, Alexander Ksendzovsky

**Affiliations:** a Department of Neurosurgery, University of Maryland School of Medicine, Baltimore, MD, USA; b Department of Pathology, University of Maryland School of Medicine, Baltimore, MD, USA; c Department of Physiology, University of Maryland School of Medicine, Baltimore, MD, USA

**Keywords:** Pentylenetetrazol, Kindling, Epilepsy, Animal model, Seizure threshold

## Abstract

Pentylenetetrazol (PTZ) kindling is a widely used model for inducing epileptogenesis and evaluating long-term seizure susceptibility differences among animals. This model is typically performed by chronic, repetitive exposures to a constant subconvulsive PTZ dose. However, the effectiveness of the commonly used dose (35 mg/kg) varies among different animal groups due to factors such as species, age, sex, and genetic background. This study characterizes a novel kindling approach, the PTZ Dose Escalation (PTZ-DE) model, which assesses chronic seizure threshold with enhanced sensitivity by empirically determining the minimally effective dose to induce PTZ kindling for specific experimental conditions. The efficacy and validity of the PTZ-DE model were compared to the standard PTZ kindling approach. First, the characteristic increase in chronic seizure response was compared between PTZ-DE and the standard model across animal characteristics (strain, sex). Next, the PTZ-DE model’s validity was assessed by determining whether PTZ-DE could replicate the increased chronic seizure susceptibility previously reported using the standard approach after traumatic brain injury (TBI). Lastly, the PTZ-DE model’s effectiveness in detecting seizure differences was measured in a condition (glyburide treatment) where alterations to chronic seizure susceptibility were not detected with standard kindling. This study observed that, compared to the standard model, the PTZ-DE model corrects for background differences in PTZ susceptibility, replicates known alterations in chronic seizure thresholds, and uncovers changes in seizure threshold previously unidentified by the standard approach. The PTZ-DE model may be a superior approach for discovering new pathological mechanisms of epileptogenesis and for developing targeted therapies for seizure management.

## 1. Introduction

Pentylenetetrazol (PTZ) kindling serves as a commonly used animal model for inducing epileptogenesis, uniquely increasing chronic seizure susceptibility without relying on status epilepticus (Pitka*nen et al., 2006; Pitkänen et al., 2007). Derived from the original electrical kindling models, which used repetitive electrical stimulations in the amygdala or hippocampus delivered by surgically implanted electrodes to gradually lower seizure threshold, the PTZ kindling model is widely used as a practical and less invasive alternative approach that similarly induces gradually worsened seizures via chronic drug administration (Dhir, 2012; Singh et al., 2021). This model is invaluable for investigating how chronic seizures impact the brain or examining how factors such as injury, pharmacological interventions, or genetic modifications affect chronic seizure susceptibility (Pitka*nen et al., 2006; Pitkänen et al., 2007).

The standard PTZ kindling model is typically performed by intraperitoneal (IP) injection of a fixed dose of PTZ (most often 30–35 mg/kg, but can range from 20 to 40 mg/kg) that is assumed to be subconvulsive every second day for either a fixed total number of days or until a “kindled” state in which animals exhibit a severe seizure phenotype after consecutive injections (Dhir, 2012; Singh et al., 2021; Shimada and Yamagata, 2018; Li et al., 2014; Mazumder et al., 2017; Samokhina and Samokhin, 2018; Erdtmann-Vourliotis et al., 1998). Repetitive exposure to this initially subconvulsive dose eventually leads to a chronically lowered seizure threshold, which results in severe seizure responses to the same low-dose PTZ exposure (Shimada and Yamagata, 2018). However, the subconvulsive nature of the chosen dose varies greatly across different animal groups or experimental conditions, as an animal’s response to PTZ is highly sensitive and is influenced by variables including species, age, sex, and genetic background (Medina et al., 2001; Aleshin et al., 2021; Grecksch et al., 1997; Löscher et al., 2017). Therefore, it is difficult to identify prior to conducting an experiment whether the chosen PTZ dose is optimal for a specific set of animals or testing conditions. To enhance the PTZ kindling model's sensitivity in detecting differences in chronic seizure susceptibility among experimental groups, and ensure the results of a given experiment are not influenced by the use of an inappropriate PTZ dose, it may be important to empirically determine the minimal effective dose to induce PTZ kindling within the context of each experiment, rather than relying on predetermined assumptions. While some researchers may preliminarily optimize dosing in a separate group of animals prior to experimentation, this approach is limited as it is resource inefficient and does not guarantee that the optimized dose will be applicable towards a new cohort of animals.

In the present study, we introduce a novel PTZ kindling approach, referred to as the PTZ Dose Escalation (PTZ-DE) model, which aims to improve the sensitivity of the PTZ kindling model to detect nuanced differences in chronic seizure susceptibility by empirically determining the optimal dose for kindling within the confines of a given experiment. Inspired by methods used in acute seizure threshold testing—which gradually increase the PTZ dose until an acute seizure occurs—this model adapts the dose escalation strategy for a precise determination of the minimally effective dose for specific experimental conditions to more effectively evaluate susceptibility to chronic seizures (Mandhane et al., 2007). In this study, we first compare this new model’s efficacy to a version of the standard fixed dosing approach, with characteristics most typically seen across PTZ kindling studies (Shimada and Yamagata, 2018), across animal background characteristics, including across sexes and genetic strains, to emulate the expected PTZ kindling response of a gradual increase in seizure phenotype. We then verify the PTZ-DE model’s capability to replicate known chronic seizure susceptibility differences observed with traditional PTZ kindling by testing the model’s performance in detecting increased seizure susceptibility to a model of traumatic brain injury (TBI). Lastly, we characterize the PTZ-DE model’s potential to identify previously undetected therapeutic effects of pharmacologic treatment by using PTZ-DE to assess the effects one drug, glyburide (GLYB), in which therapeutic effects of treatment are not observed with standard PTZ kindling.

## 2. Materials and methods

### 2.1. Ethics statement

Animal experiments comply with the ARRIVE guidelines and were performed under a protocol approved by the Institutional Animal Care and Use Committee of the University of Maryland School of Medicine, and in accordance with the relevant guidelines and regulations as stipulated in the United States National Institutes of Health Guide for the Care and Use of Laboratory Animals.

### 2.2. Subjects

C57/Bl6N mice were males and females aged 6–8 weeks obtained from Charles River Laboratories, whereas C57/Bl6N+ 129/SvJ mice were male and female mice aged 10–16 weeks and obtained from our breeding colony. For sex comparison experiments, both males and females were C57/Bl6N mice aged 6–8 weeks. For TBI experiments, C57/Bl6N mice were obtained from Envigo and were 8–12-week-old males. GLYB and vehicle (VEH) treated C57/Bl6N mice were male and female aged 6–8 weeks old obtained from Charles River Laboratories.

### 2.3. Drug administration

PTZ (Millipore-Sigma, P6500) was reconstituted in 1X PBS to a stock concentration of 10 mg/ml and syringe filtered for sterility, after which PTZ was administered to mice via IP injection. For GLYB experiments, 10 mg of GLYB (Millipore-Sigma, G2539) was weighed and reconstituted in 1 ml of 100 % corn oil (VEH) and diluted to 0.3 mg/ml, after which 100 μL of GLYB (final dose of 30 μg/mouse) was administered IP daily concurrently with PTZ treatments. On PTZ administration days, GLYB was given 15 min prior to PTZ injection.

### 2.4. Seizure phenotype scoring

Seizure severity scoring was performed for 30 min following all PTZ injections as previously described using a modified version of the Racine scoring scale (Racine, 1972; Lüttjohann et al., 2009; Moyer et al., 2025). The following scoring classifications were used: 0. No seizure response, 1. Behavioral arrest or slowing, 2. Head nodding associated with facial clonus, 3. Partial limb clonus with or without tail stiffening, 4. Clonic seizure with rearing and loss of posture, 5. Generalized tonic-clonic seizure with wild running and/or jumping, 6. Death. After death, mice were assigned a severity score of 6 for each day for the remainder of the experiment to prevent sampling bias and maintain statistical power between groups for the entire experiment.

### 2.5. Standard PTZ kindling

Standard PTZ kindling was induced as previously described (Shimada and Yamagata, 2018). In brief, PTZ (35 mg/kg, IP) was administered to mice every second day for a total of twenty days, i.e. 10 total PTZ administrations. Most mice reached a “fully kindled” state, defined as at least 3 responses of seizure score 4 +, by the end of PTZ kindling induction.

### 2.6. PTZ-DE kindling

**Fig. 1.**
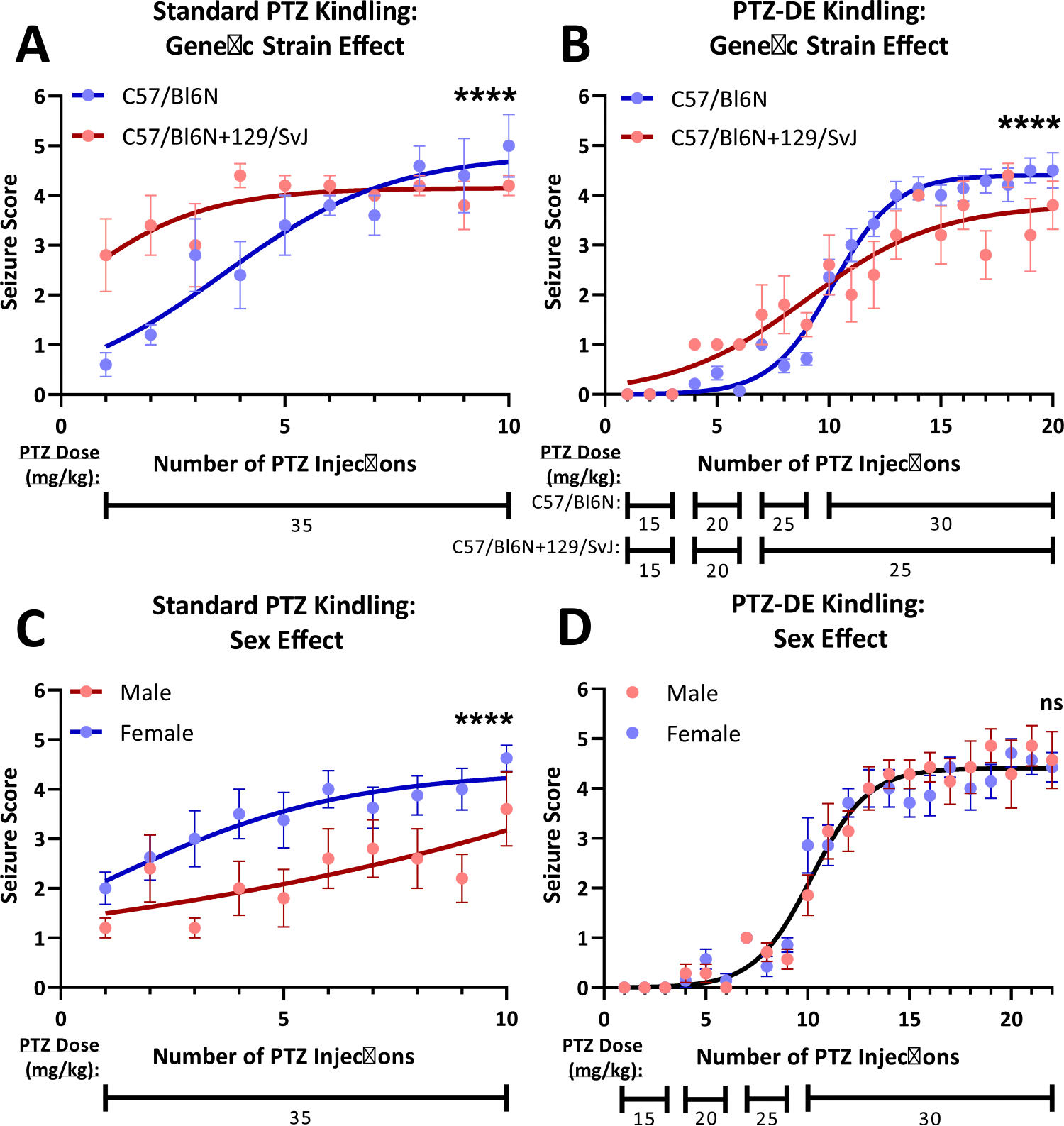
PTZ-DE Model Determines the Minimally Effective PTZ Dose to Correct Variabilities in PTZ kindling. A) Mice bred on a C57Bl/6 N + 129/SvJ background (n = 5) exhibit elevated initial response compared to C57/Bl6N mice (n = 5) in standard PTZ kindling. B) In a separate experiment, the PTZ-DE model empirically determines the true minimally effective dose to recreate gradually increasing seizure response to PTZ over the course of induction. C57Bl/6N mice (n = 14) optimally kindle at 30 mg/kg, whereas C57Bl/6 N + 129/SvJ mice (n = 5) kindle at 25 mg/kg. C) C57/Bl6N female mice (n = 8) demonstrate heightened response to standard PTZ kindling compared to males (n = 5). D) In a separate experiment, when kindled using PTZ-DE up to 30 mg/kg, no difference in male (n = 7) and female (n = 7) responses is seen. ns: not significant, ****p < 0.0001.

To evaluate differences in chronic seizure susceptibility, we modified the standard PTZ kindling model by initiating with a lower starting dose of 15 mg/kg administered IP and incrementally increasing this dose by 5 mg/kg after every three administrations. This escalation continued until at least one animal from any group exhibited a score 4 or higher seizure response. Following this, the dose that elicited a seizure score 4 + response in was consistently used for subsequent PTZ administrations in all animals within an experiment irrespective of experimental group until group responses stabilized near a “fully kindled” state (seizure score 4 + for at least 3 doses). This means that all animals in all experimental groups receive administration, once it is determined, of the same minimal dose sufficient to induce a score 4 + seizure in a single animal within the cohort of animals being used in a given experiment. By utilizing this approach, the PTZ-DE model empirically calibrates the dynamic range for detection of seizure differences between groups to the specific cohort of animals being assessed, with the lower boundary of this range being the most sensitive animal in the cohort and the upper boundary as the point when most animals have achieved “fully kindled” status. For the experiments assessing strain or sex specific PTZ responses (Fig. 1B, D), the minimally convulsive dose (i.e. dose until first seizure observed) was determined for each group, rather than applying the minimal dose across both groups, to demonstrate the different effective dose across background characteristics. PTZ administrations were still conducted every other day, as in the standard protocol, but with a variable total number of doses administered, typically ranging from 20 to 25.

### 2.7. Controlled cortical impact (CCI) induction

Mice underwent a controlled cortical impact (CCI) to model moderate to severe traumatic brain injury (TBI) (Tata et al., 2021). Briefly, mice were anesthetized with 4 % isoflurane in oxygen and placed in a stereotactic frame (Stoelting Co., 51615). A left scalp incision was made. A dental drill was used to make a 5-mm craniotomy over the left parietal cortex, and the bone flap was removed. CCI was performed with an electromagnetic CCI impactor (Leica Biosystems, 39463920) with a flat tipped 3-mm diameter impounder on the left parietal cortex (velocity=1 m/s, depth = 2.5 mm, dwell time = 200 ms). Mice recovered in a temperature-controlled chamber before returning to their cage. Sham control mice underwent the identical procedure up until removal of bone; however, no impaction was induced. PTZ-DE was initiated 7 days after CCI.

### 2.8. Statistical analysis

**Fig. 2.**
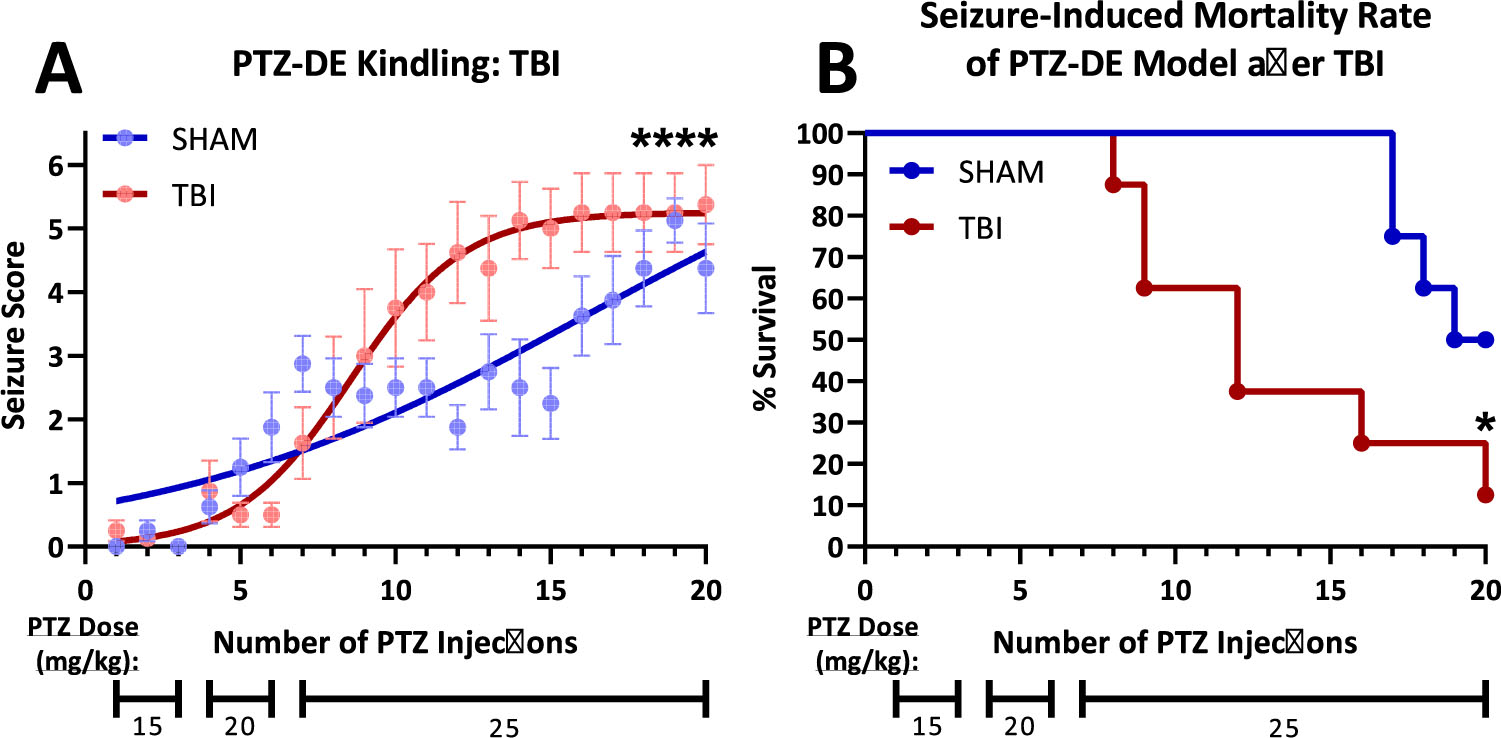
The PTZ-DE Model Replicates Elevated PTZ Kindling Response After TBI A) TBI induced mice (n = 8) develop more severe seizure responses with fewer PTZ administrations in the PTZ-DE model compared to sham controls (n = 8). B) Kaplan-Meier analysis demonstrating increased mortality due to PTZ induced seizures in TBI mice (n = 8) compared to sham controls (n = 8) when assessed via PTZ-DE. *p < 0.05, ****p < 0.0001.

**Fig. 3.**
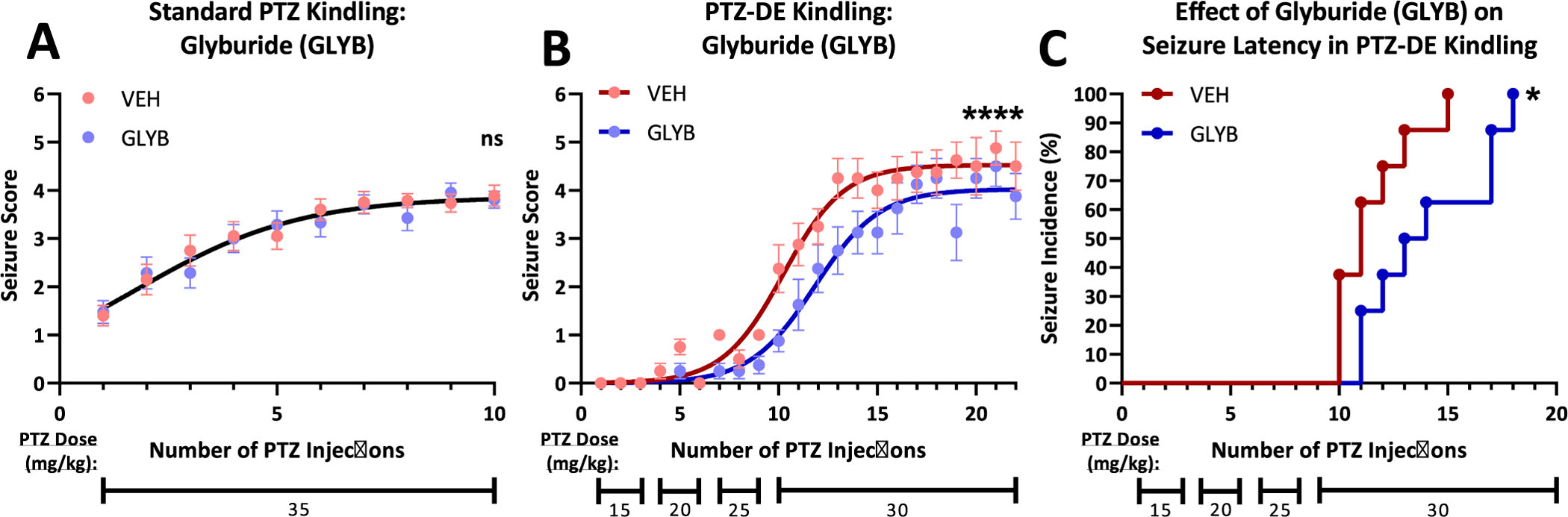
The PTZ-DE Model Improves the Detection Sensitivity of PTZ Kindling to Detect Underlying Differences in Chronic Seizure Susceptibility. A) Mice undergoing concurrent daily glyburide (GLYB) administration (n = 20) demonstrate similar seizure response to standard PTZ kindling compared to vehicle controls (n = 20). B) Mice undergoing concurrent daily GLYB administration (n = 8) with PTZ-DE kindling demonstrate significantly less severe seizure response compared to vehicle control (n = 8) mice. C) Daily GLYB treatment increases the number of PTZ administration in the PTZ-DE model required to induce the first severity 4 + (tonic-clonic) seizure response. ns: not significant *p < 0.05, ****p < 0.0001. B has been reproduced from Moyer, et. al., 2025 (Moyer et al., 2025).

Data are presented as mean ± SEM unless noted otherwise. Non-linear regression analyses using a logistic growth model with an extra sum-of-squares F test were used to assess for significant differences between PTZ kindling severity curves. Logistic growth was chosen as the most appropriate model for all experiments (excluding Kaplan-Meier analyses), as the sigmoidal curve inferred when using a logistic growth model best represents the expected phases of PTZ kindling, which include the initial minimal responsive to PTZ due to the subconvulsive nature of the dose, followed by the growth phase in which mice begin exhibiting a seizure response to PTZ, and finally the plateau phase in which mice have fully kindled (Shimada and Yamagata, 2018). To assess which initial responses (Y_0_) were closest to zero, Y_0_ values were compared between curves by applying Akaike’s Information Criterion (AICc) to evaluate two candidate models: one with the setting 0 <y0 > 0.2 and one with Y_0_≥ 0.2. The model with the smaller AICc value is statistically preferred. Growth rate constants and maximal values (Ymax) were assessed by logistic growth model with an extra sum-of-squares F test for Figs. 2 and 3 respectively. Additionally, R^2^ analysis was used to assess how well the logistic growth model fits the data. from Log-rank tests were performed to assess statistical differences between Kaplan-Meier curves. A complete summary of statistical analyses and their resulting descriptive statistics can be found in Supplemental Table 1. Analyses were performed with GraphPad Prism 9.3.1. P < 0.05 was deemed to be statistically significant.

## 3. Results

### 3.1. PTZ-DE better models PTZ kindling through empirical subconvulsive dose determination

To determine whether the PTZ-DE model can identify alterations in seizure susceptibility to PTZ caused by background differences among mice, we directly compared the responses of mice subjected to PTZ-DE with those treated using the standard PTZ protocol. In an initial assessment of chronic seizure susceptibility using the standard PTZ kindling model, C57/Bl6N mice (n = 5) were compared to C57/Bl6N+ 129/SvJ mice (n = 5), as 129/SvJ strain mice are commonly used for breeding genetic knockout mouse lines (Bothe et al., 2005; White et al., 2002). Compared to C57/Bl6N mice (Y_0_=0.616), mice bred on a C57/Bl6N+ 129/SvJ background exhibited significantly elevated initial seizure responses (Y_0_=2.076, p < 0.001; F=8.964) to the first 35 mg/kg dose of PTZ, with sustained high seizure severity scores throughout the experiment (Fig. 1A). This unexpected response suggested that the conventional 35 mg/kg dose may not accurately capture the gradual increase in seizure severity that PTZ kindling aims to model, where the initial response (Y_0_) should be close to 0 across all conditions and progressively elevate over the course of the kindling process. Consequently, we used the PTZ-DE model to determine and compare the true minimally effective dose for these genetic backgrounds (Fig. 1B). To ensure the minimal dose was captured, we started with 15 mg/kg—significantly lower than typically reported in PTZ kindling literature-and incrementally increased the dose after every three administrations until a severity score 4 + seizure response was observed in at least one mouse from either group. After the first severity score 4 + seizure, this empirically determined minimally effective dose was maintained for the rest of the experiment. A score of 4 in at least one animal was used to define the appropriate PTZ dose because this represents the point where at least one animal reached a “kindled” state characterized by development of a full tonic-clonic seizure rather than a single convulsive episode. Once the first animal exhibits a kindled response to the empirically determined dose, it can be reasonably expected that the other animals with a given background will also appropriately respond to kindling, thus that dose is continued for the remainder of the experiment. Both C57/Bl6N (n = 14) and C57/Bl6N+ 129/SvJ (n = 5) mice appropriately displayed minimal seizure responses (Score 0–1) (C57/Bl6N: Y_0_=0.0029; C57/Bl6N+SvJ: Y_0_=0.1681) at 15 mg/kg and 20 mg/kg PTZ, but C57/Bl6N+ 129/SvJ mice began to show increasing severity scores from 25 mg/kg onwards, whereas C57/Bl6N mice only began exhibiting severity score 4 + seizure responses after reaching 30 mg/kg. These observations led to the conclusion that the most appropriate PTZ dose for kindling C57/Bl6N+ 129/SvJ mice is 25 mg/kg, which is significantly lower than the standard 35 mg/kg dose, and 30 mg/kg for C57/Bl6N mice. This finding explains the initially severe response of C57/Bl6N+ 129/SvJ mice compared to C57/Bl6N mice under the standard model.

In addition to identifying the true minimally effective dose for kindling, the PTZ-DE model more accurately replicated the characteristic pattern of PTZ kindling, marked by an initial minimal response, a gradual increase in seizure severity and eventual plateau (Supplemental Table 1). When fitted to the logistic growth model, which represents the characteristic response of PTZ kindling, PTZ-DE curves demonstrated improved measures of fit compared to the standard model (PTZ-DE: R^2^: C57/Bl6N: 0.8262, C57/Bl6N+129/SvJ: 0.6366; standard: R^2^: C57/Bl6N: 0.5826, C57/Bl6N+SvJ: 0.1744). Furthermore, the initial responses of both strains were appropriately near zero in the PTZ-DE models (C57/Bl6N: Y_0_=0.0029; C57/Bl6N+129/SvJ: Y_0_=0.1681, AICc analysis preferred the 0<Y_0_<0.2 setting models for both strains), whereas mice undergoing standard kindling exhibited elevated initial responses (C57/Bl6N: Y_0_=0.616; C57/Bl6N+129SvJ: Y_0_=2.076, AICc analysis preferred the Y_0_≥0.2 setting models for both strains). Together, these data suggest that the PTZ-DE model more accurately replicates the expected PTZ kindling response, especially during the early phase of kindling, compared to the standard model.

Although the PTZ-DE model reduced the group variability in PTZ response due to genetic strain differences, and more closely produced the expected gradual increase in seizure severity than standard PTZ kindling, the PTZ-DE kindling curves exhibited by C57/Bl6N and C57/Bl6N+ 129/SvJ mice were still significantly different (p < 0.001, F=14.47) due to the differing minimally effective doses by each genetic strain (Fig. 1B). To that end, we tested whether the PTZ-DE model could reduce differences in another background characteristic - sex - seen in standard PTZ kindling (Fig. 1C). Male C57/Bl6N mice (n = 5) undergoing standard PTZ kindling demonstrated a significantly decreased (p < 0.001, F=10.86) kindling response compared to female C57/Bl6N mice (n = 8). Additionally, while female mice achieved full kindled status as a group (Ymax: 4.368) during standard kindling, male mice failed to fully kindle within the timeframe of the kindling protocol. This is further supported by the infinitely high (unstable) Ymax value determined by the logistic growth curve, indicating that the male group response had not yet plateaued (Supplemental Table 1). Using the PTZ-DE model, male and female mice exhibited comparable responses (p = 0.235, F=1.427), characterized by minimal seizure phenotypes (Score 0–1) until a PTZ dose of 30 mg/kg, after which both sexes (n = 7 for each sex) began to develop severe seizure responses at similar rates (Fig. 1D). Both groups ultimately achieved fully kindled status (Ymax: Male: 4.544; Female: 4.265). Furthermore, as observed with genetic strains, PTZ-DE curves for both sexes demonstrated improved fit with the logistic growth model (R^2^: Male: 0.8158; Female: 0.8436) and a lower initial starting point (Y_0_: Male: 0.002685; Female: 0.002089, AICc analysis preferred the 0 <Y_0_<0.2 setting models for both sexes) compared to the standard model (R^2^: Male: 0.1670, Female: 0.2508; Y_0_: Male: 1.374; Female: 1.744, AICc analysis preferred the Y_0_≥0.2 setting models for both sexes) (Supplemental Table 1). These findings suggest that the PTZ-DE model can account for some differences in PTZ kindling responses due to animal background characteristics and further supports the notion that PTZ-DE induces a more accurate kindling response.

### 3.2. PTZ-DE detects chronic seizure threshold differences previously identified by standard PTZ kindling

To validate the efficacy of the PTZ-DE model in detecting differences in chronic seizure susceptibility, we used dose escalation in a scenario where such differences in chronic seizure responses are expected and have been previously described in standard kindling: TBI induced by a CCI model. Previous studies using standard PTZ kindling methods had demonstrated reduced seizure thresholds in CCI mice compared to sham controls (Carver et al., 2021; Eslami et al., 2015, 2016). Using the PTZ-DE kindling approach, we assessed whether it could similarly detect alterations in seizure responses induced by CCI, thereby testing the validity of the PTZ-DE model. We observed that mice in both groups showed minimal responses to PTZ doses of 15 mg/kg or 20 mg/kg; however, at 25 mg/kg, CCI mice (n = 8) exhibited significantly greater seizure responses compared to sham controls (n = 8) (p < 0.001, F=16.29), characterized by a substantially faster elevation in scores (Growth Rate Constant (GRC): Sham: 0.1462, TBI: 0.5483; p = 0.001, F= 10.80, extra sum-of-squares F test) (Fig. 2A, Supplemental Table 1). The minimal response observed until the 25 mg/kg dose indicates that this was the appropriate minimally effective PTZ dose for kindling in our TBI model. Additionally, the severe response in CCI mice was accompanied by a higher mortality rate due to seizures (p = 0.0297) compared to sham control mice (Fig. 2B). Together, these results obtained using the PTZ-DE model align with prior findings from models using the standard PTZ model (Carver et al., 2021; Eslami et al., 2015, 2016), corroborating both the efficacy of PTZ-DE model in detecting differences in chronic seizure susceptibility and previous evidence that TBI lowers the chronic seizure threshold.

### 3.3. PTZ-DE enhances sensitivity for detecting chronic seizure susceptibility differences compared to standard PTZ kindling

To directly compare the PTZ-DE model's ability to detect nuanced differences in seizure susceptibility due to pharmacological interventions to the standard kindling approach, we used both the models alongside concurrent daily administration of GLYB, an inhibitor of the SUR1-TRPM4 channel. This channel is thought to have protective effects against chronic seizure development (Lin et al., 2017; Chen et al., 2020; Mundrucz et al., 2023). We selected GLYB treatment for this comparison, rather than using a standard antiseizure medication, which would affect standard PTZ kindling, because preliminary experiments showed no response to GLYB treatment in the standard model, unlike its effects on other epilepsy models (Lin et al., 2017). Therefore, we aimed to determine whether the PTZ-DE model could detect antiseizure effects of GLYB treatment on chronic seizure susceptibility that were missed by the standard approach. In the standard PTZ kindling setup, concurrent GLYB treatment (n = 20) did not alter kindling rates compared to vehicle control treatment (n = 20) (p = 0.919, F=0.1671) (Fig. 3A). However, with the PTZ-DE model, mice co-treated with GLYB (n = 8) exhibited a significantly reduced seizure response compared to controls (n = 8) (p < 0.001, F=15.29), characterized by a rightward shift in the kindling response curve and decreased scores throughout, including the maximal plateau (Ymax: VEH: 4.527; GLYB: 4.025, p = 0.03, F=4.927, extra sum-of-squares F-test) (Supplemental Table 1). Additionally, GLYB-treated mice undergoing the PTZ-DE model required significantly more 30 mg/kg PTZ administrations to achieve score 4 or above seizures (p = 0.04) compared to vehicle treated mice (Fig. 3C). As with the experiments in 3.1, PTZ-DE kindling curves demonstrated a better fit with the logistic growth model (R^2^: VEH: 0.7957; GLYB: 0.7404) compared to standard kindling curves (R^2^: VEH: 0.3334; GLYB: 0.2792). These findings indicate that the PTZ-DE model offers enhanced sensitivity to detect subtle chronic seizure threshold changes in response to pharmacologic treatment compared to the traditional PTZ kindling approach.

## 4. Discussion

The PTZ kindling method serves as a reliable model for assessing chronic seizure susceptibility, independent of status epilepticus. In this study, we introduce a novel PTZ-DE model which employs progressively increasing doses of PTZ to empirically identify the lowest convulsive dose required for kindling under varying experimental conditions (Fig. 1). This approach not only replicates the effects observed with the standard PTZ kindling model (Fig. 2) but also enhances the detection of differential responses across diverse applications (Fig. 3). Consequently, the PTZ-DE model may offer enhanced translational potential by improving the ability to identify nuanced differences in genetic or pharmacological therapies, thereby uncovering novel therapeutic targets and minimizing the risk of missing opportunities for preventing or treating epilepsy.

The PTZ-DE model improves on the traditional PTZ kindling approach by offering greater sensitivity and applicability across a broader range of animals and experimental conditions. Variability in group responses to PTZ, influenced by factors such as age, sex, and genetic background, poses challenges in the standard PTZ kindling model, which assumes uniform responses to a fixed PTZ dose (Medina et al., 2001; Aleshin et al., 2021; Grecksch et al., 1997; Löscher et al., 2017). This assumption can introduce variability between groups when using animals with diverse characteristics, such as genetic knockout models (Fig. 1). By tailoring PTZ doses to the specific attributes of animals in each experiment, the PTZ-DE model improves the sensitivity of PTZ kindling to detect subtle differences in chronic seizure susceptibility (Figs. 1, 3). It is important to note, however, that while the PTZ-DE model reduces variations in response due to background characteristics, as is evidenced by decreased sex and genetic strain differences in PTZ kindling response compared to the standard model (Fig. 1B), proper control of animal background characteristics within an experiment should still be maintained to ensure any differences in chronic seizure threshold detected by the PTZ-DE model are due to the experimental treatment. Additionally, the PTZ-DE model improves sensitivity by increasing the total number of PTZ administrations, thereby enhancing degrees of freedom and statistical power (Supplemental Table 1). Importantly, by accounting for variations in seizure responses caused by diverse genetic backgrounds by tailoring the PTZ kindling dose to a specific animal cohort’s background, the PTZ-DE model offers improvements in experiments using genetic knockout models bred on mixed backgrounds.

Beyond improving sensitivity, the PTZ-DE model also better replicates the expected kindling response compared to standard PTZ kindling, ideally represented by an S-shaped curve modeled through logistic growth regression (Fig. 1, Supplemental Table 1). As demonstrated throughout our data, PTZ-DE curves consistently maintain a better fit to the observed data (represented by R^2^ and RMSE values) compared to standard PTZ curves (Supplemental Table 1). Starting with a lower PTZ dose followed by gradual escalation, the PTZ-DE model produces minimal seizure responses (Score 0) at the beginning of kindling (represented in the logistic growth model by a low Y_0_ value) until the minimal effective dose is reached (Fig. 1, Supplemental Table 1). Once reached, seizure severity increases and eventually plateaus (Ymax) as the “fully kindled” state is reached (Fig. 1, Supplemental Table 1). In contrast, the standard PTZ kindling model exhibits an elevated Y_0_ values due to the typically used starting dose of 35 mg/kg, which is higher than the minimally effective dose in most cases (Fig. 1, Supplemental Table 1). This elevated initial response limits the ability to model the chronic lowering of seizure thresholds—a key feature of epileptogenesis. The PTZ-DE model overcomes this issue by empirically determining the minimally effective dose, ensuring the starting dose for kindling is at or below that dose and accurately captures the phase of chronic seizure threshold reduction.

An alternative approach used in some studies to empirically determining the optimal PTZ dose is to conduct a series of pilot studies to establish a PTZ dose response curve, with either acute or chronic PTZ exposure, and using this to inform a standard PTZ kindling protocol (e.g., targeting an EC25 point). However, this approach does not account for cohort variability in PTZ response and would be comparatively time and cost prohibitive, especially in conditions where breeding is required for experiments. The PTZ-DE model is a superior approach because it determines the ideal PTZ dose for a given experiment not only within varied animal backgrounds, but also within the exact set of animals being used for a given experiment. This is cost-effective and eliminates the effect of cohort variability in response to PTZ.

Despite its strengths, a limitation of PTZ kindling, and thus the PTZ-DE model, is the lack of consistent spontaneous seizures, a hallmark of epilepsy in patients (Fisher et al., 2014). Alternative chemical induction models such as lithium pilocarpine or kainic acid, which reliably produce spontaneous seizures are often used (Löscher, 2002). However, these models require inducing status epilepticus and/or directly injecting the brain, complicating the distinction between chronic epilepsy mechanisms and those resulting from primary injuries like status epilepticus or trauma (Löscher, 2002). These limitations narrow their translational applicability (Langenbruch et al., 2021). In contrast, PTZ-DE kindling simulates chronic epileptogenesis without requiring a primary injury, making it broadly applicable to a variety of chronic epilepsies (Shimada and Yamagata, 2018; Löscher, 2002).

The primary limitation of the present study is the absence of electrographic characterization of the PTZ-DE model, which we intentionally avoided to prevent confounding effects from intracranial electrode implant (Campbell and Wu, 2018). This omission means potential unique electrographic events or spontaneous seizures induced by this modified kindling approach may have been missed. Given the standard PTZ models known limitation in reliably producing spontaneous seizures, future studies should incorporate long-term electrographic monitoring of the PTZ-DE model. This would clarify whether the increased number of PTZ administrations elevates the model's severity and potentially induces spontaneous seizure activity. An additional limitation was that ovarian cycles for female mice were not tracked during induction of either standard PTZ kindling or the PTZ-DE model, which may have provided further information on the nature of PTZ kindling responses in female mice. Future studies aimed at characterizing differences in female mouse seizure response to PTZ-DE kindling based on estrous cycle stage would provide additional insights into the effects that fluctuations in sex hormones have on chronic seizure responses.

## 5. Conclusions and future directions

The PTZ-DE model is a novel method designed to evaluate changes in chronic seizure thresholds, building upon traditional PTZ kindling by incorporating dose escalation, a concept adapted from acute seizure threshold testing. This model enhances sensitivity in detecting differences in seizure susceptibility across various conditions and allows for customization of PTZ dosing tailored to specific experimental conditions. As a result, the PTZ-DE model has the potential to facilitate the discovery of new therapeutic targets and personalized treatments for seizure management and prevention in epilepsy.

## Supplementary Material

1

## Data Availability

All data are available in the main text or the supplementary materials. All reported data will be shared by the corresponding author upon request.

## Appendix A. Supporting information

Supplementary data associated with this article can be found in the online version at doi:10.1016/j.eplepsyres.2026.107755.

## Abbreviations:

PTZ: Pentylenetetrazol
PTZ-DE: Pentylenetrazol Dose Escalation
IP: Intraperitoneal
TBI: Traumatic Brain Injury
GLYB: Glyburide
VEH: Vehicle
CCI: Controlled Cortical Impact
AICc: Akaike’s Information Criterion
GRC: Growth Rate Constant
