# Supplementary material for: Dose escalation in pentylenetetrazol kindling detects differences in chronic seizure susceptibility
Source: Epilepsy Res. Author manuscript; Available in PMC 2026 Jun 23. (PMC13288531; doi:10.1016/j.eplepsyres.2026.107755)
Supplement: 1 [file NIHMS2178258-supplement-1.docx]

| **Supplemental Table 1: Complete Statistical Description for All Comparisons in This Study** | | | | | | | | **Effect Size** | | | **Variability/Fit Measure** |  |
| --- | --- | --- | --- | --- | --- | --- | --- | --- | --- | --- | --- | --- |
| **Figure** | **Comparison** | **Sample Size** | **Test Used** | **Conclusion/Interpretation** | **P-Value** | **F-Value** | **Degrees of Freedom** | **Growth Rate Constant (k)** | **Y_0_** | **Ymax** | **R^2^** | **Chi-Square** |
| 1A | Genetic Strain (C57/Bl6N vs C57/Bl6N+SvJ) PTZ Standard | C57/Bl6N: 5 C57/Bl6N+129/SvJ: 5 | Logistic Growth, Least Square Fit | Reject the null hypothesis; Different curve for each data set | <0.0001 | 8.964 | C57/Bl6N: 47 C57/Bl6N+129/SvJ: 47 | C57/Bl6N: 0.5319 C57/Bl6N+129/SvJ: 0.6684 | C57/Bl6N: 0.6160 C57/Bl6N+129/SvJ: 2.076 | C57/Bl6N: 4.835 C57/Bl6N+129/SvJ: 4.152 | C57/Bl6N: 0.5826 C57/Bl6N+129/SvJ: 0.1744 | N/A |
| 1B | Genetic Strain (C57/Bl6N vs C57/Bl6N+SvJ) PTZ-DE | C57/Bl6N: 14 C57/Bl6N+129/SvJ: 5 | Logistic Growth, Least Square Fit | Reject the null hypothesis; Different curve for each data set | <0.0001 | 14.47 | C57/Bl6N: 305 C57/Bl6N+129/SvJ: 97 | C57/Bl6N: 0.7196 C57/Bl6N+129/SvJ: 0.3419 | C57/Bl6N: 0.002903 C57/Bl6N+129/SvJ: 0.1681 | C57/Bl6N: 4.409 C57/Bl6N+129/SvJ: 3.814 | C57/Bl6N: 0.8262 C57/Bl6N+129/SvJ: 0.6366 | N/A |
| 1C | Sex (M vs F) PTZ Standard | Male: 5 Female: 8 | Logistic Growth, Least Square Fit | Reject the null hypothesis; Different curve for each data set | <0.0001 | 10.86 | Male: 57 Female: 77 | Male: 0.08362 Female: 0.3765 | Male: 1.374 Female: 1.744 | Male: 3.717E+86 (unstable) Female: 4.368 | Male: 0.1670 Female: 0.2508 | N/A |
| 1D | Sex (M vs F) PTZ-DE | Male: 7 Female: 7 | Logistic Growth, Least Square Fit | Do not reject the null hypothesis; One curve for all data sets | 0.2350 | 1.427 | Male: 151 Female: 151 | Male: 0.7096 Female: 0.7724 | Male: 0.002685 Female: 0.002089 | Male: 4.544 Female: 4.265 | Male: 0.8158 Female: 0.8436 | N/A |
| 2A | SHAM vs TBI PTZ-DE Racine | SHAM: 8 TBI: 8 | Logistic Growth, Least Square Fit | Reject the null hypothesis; Different curve for each data set | <0.0001 | 16.29 | SHAM: 157 TBI: 157 | SHAM: 0.1462 TBI: 0.5483 | SHAM: 0.6277 TBI: 0.04763 | SHAM: 7.265 TBI: 5.253 | SHAM: 0.4382 TBI: 0.5979 | N/A |
| 2B | SHAM vs TBI PTZ-DE Mortality | SHAM: 8 TBI: 8 | Log-rank (Mantel-Cox) test | Reject the null hypothesis | 0.0297 | N/A | N/A | N/A | N/A | N/A | N/A | 4.726 |
| 3A | VEH vs GLYB PTZ Standard | VEH: 20 GLYB: 20 | Logistic Growth, Least Square Fit | Do not reject the null hypothesis; One curve for all data sets | 0.9185 | 0.1671 | VEH: 194 GLYB: 207 | VEH: 0.5739 GLYB: 0.4901 | VEH: 1.037 GLYB: 1.150 | VEH: 3.861 GLYB: 3.874 | VEH: 0.3334 GLYB: 0.2792 | N/A |
| 3B | VEH vs GLYB PTZ-DE Racine | VEH: 8 GLYB: 8 | Logistic Growth, Least Square Fit | Reject the null hypothesis; Different curve for each data set | <0.0001 | 15.29 | VEH: 173 GLYB: 173 | VEH: 0.6525 GLYB: 0.6089 | VEH: 0.005675 GLYB: 0.002982 | VEH: 4.527 GLYB: 4.025 | VEH: 0.7957 GLYB: 0.7404 | N/A |
| 3C | VEH vs GLYB PTZ-DE Seizure Latency | VEH: 8 GLYB: 8 | Log-rank (Mantel-Cox) test | Reject the null hypothesis | 0.036 | N/A | N/A | N/A | N/A | N/A | N/A | 4.398 |
